# Physiological, emotional and neural responses to visual stimuli in eating disorders: a review

**DOI:** 10.1186/s40337-021-00372-1

**Published:** 2021-02-17

**Authors:** Victoria Burmester, Esme Graham, Dasha Nicholls

**Affiliations:** Division of Psychiatry, Department of Brain Sciences, Imperial College London, Du Cane Road, London, W12 0NN UK

**Keywords:** Emotional, Physiological, Neuroimaging, Anorexia nervosa, Bulimia nervosa, Binge eating disorder, Shape/weight, Food, Visual stimuli, Images

## Abstract

**Background:**

Overconcern with food and shape/weight stimuli are central to eating disorder maintenance with attentional biases seen towards these images not present in healthy controls. These stimuli trigger changes in the physiological, emotional, and neural responses in people with eating disorders, and are regularly used in research and clinical practice. However, selection of stimuli for these treatments is frequently based on self-reported emotional ratings alone, and whether self-reports reflect objective responses is unknown.

**Main body:**

This review assessed the associations across emotional self-report, physiological, and neural responses to both food and body-shape/weight stimuli in people with anorexia nervosa (AN), bulimia nervosa (BN) and binge eating disorder (BED). For food stimuli, either an aversive or lack of physiological effect was generated in people with AN, together with a negative emotional response on neuroimaging, and high subjective anxiety ratings. People with BN showed a positive self-rating, an aversive physiological reaction, and a motivational neural response. In BED, an aversive physiological reaction was found in contrast to motivational/appetitive neural responses, with food images rated as pleasant. The results for shape/weight stimuli showed aversive responses in some physiological modalities, which was reflected in both the emotional and neural responses, but this aversive response was not consistent across physiological studies.

**Conclusions:**

Shape/weight stimuli are more reliable for use in therapy or research than food stimuli as the impact of these images is more consistent across subjective and objective responses. Care should be taken when using food stimuli due to the disconnect reported in this review.

## Plain English summary

Images of food and body shape are extremely salient to people with eating disorders (ED). These images cause changes to occur in physiological measures, such as heart rate, and in neural function, as seen on brain scans. Because such images are used in clinical practice, it is important that they induce reliable physiological and neural responses. However, images used in treatment are usually selected based on patients’ subjective emotional ratings or by clinicians using variable criteria. It is important to know how well patients’ subjective ratings correlate with physiological and neural measures.

This review looks at the responses of people across ED diagnoses (anorexia nervosa, bulimia nervosa and binge-eating disorder) and compares subjective ratings, physiological and neural responses to food and body shape images. For food stimuli, emotional responses were mixed, but overall emotional ratings for these images did not reflect the changes that were seen physiologically or in neuroimaging. Images of body shapes resulted in participants providing negative emotional ratings, with physiological and neural measures also reflecting negative emotion.

This suggests that body shape images are potentially more useful in clinical practice and research than food images, as emotional ratings better represent the changes seen both in brain scans and physiological tests.

## Background

Eating disorders (ED) are defined by negative thoughts and behaviours towards eating, body-shape and weight, leading to disturbance of eating behaviour. The main ED diagnoses are anorexia nervosa (AN), bulimia nervosa (BN) and binge eating disorder (BED) [1, 2]. The transdiagnostic model of ED suggests that overconcern with shape, weight and food is central to maintenance of all ED and that clinical features are maintained by common underlying psychopathology, including food, weight, and shape overconcern [3].

The impact of overconcern is manifest as attentional bias. Beck and Clark’s information-processing model reveals the importance of attentional biases for information that is seen as threatening to the individual; this is thought to be the basis of anxiety [4]. Such attentional bias has been shown to be present in those with ED [5], effects that differ in healthy controls, reinforcing the role of salient visual stimuli in ED psychopathology. Looking at attentional bias to a range of stimuli is key to understanding the effects of visual stimuli on those with ED pathology and its translation into physiological, emotional, and neural responses. Understanding these responses can help inform clinical practice involving imagery, such as cognitive behavioural therapy (CBT) and virtual reality (VR) based treatments [6, 7].

Attentional bias has been measured using a range of different tasks. The Stroop Colour and Word Test (Stroop) requires participants to read a list of colours printed in incongruent coloured ink, such that the word “red”, might be coloured blue, and subsequent delay in colour-naming constitutes attentional bias [8]. Performance on the Stroop by women with ED showed they were slower to name colour words related to food and body image than healthy controls [9, 10]. This was replicated for food words in those with AN and BN, who were slower naming food than neutral words and slower than the control group [11]. These early findings have been extended using the dot probe, which involves participants looking at two images or words on a screen and responding as quickly as possible to a probe that appears in place of one; the response time indicates potential bias [12]. Rieger et al. found a bias towards words connoting a large physique and away from those representing a small physique in those with ED [13]. For eating, weight, and shape images, patients with ED were quicker to respond to the probe when it appeared in the same location as negative eating and neutral weight stimuli, indicating an attentional bias is present to these stimuli [5]. No effect, though, was found for shape stimuli [5]. However, in those with AN, attentional bias towards shape stimuli is found when an image is shown of the participant’s own body but did not produce attentional bias in participants with BN, suggesting attentional biases differ among ED diagnoses [14]. The spatial cueing task is similar to the dot probe; a cue picture is presented on one side of a fixation cross; a dot then appears on the same or opposite side of the picture that the participant must respond to [15]. Participants with BED preferentially identified food words in a clarification task [16] faster than weight-matched controls, and also demonstrated attentional bias to food stimuli via a stimulus engagement effect [17]. This bias was heightened when viewed in the context of real-world stimuli, with BED participants focusing gaze on food items significantly more than controls [18].

The strong attentional bias demonstrated by these studies emphasises the importance of visual stimuli for those with ED pathology and reinforces its current use in therapeutic practice. However, as highlighted by Giel et al.’s review of responses to pictorial food stimuli in those with ED, there is not always consistency for food stimuli in the responses seen and there is often a disconnect between subjective and objective responses [19]. The present review builds on the previous findings of Giel et al. by including new papers looking at food stimuli and additionally assesses responses to body shape stimuli that have been shown to be important in those with ED, to provide further clarity on relationships that exist across these measures of emotion.

Emotions involve a range of processes including affective, cognitive, and physiological responses, so these different aspects should be considered together [20]. The processing of emotions alters physiological states via the autonomic nervous system, with activity in the anterior cingulate and amygdala associated with generating a physiological response to emotions [21]. This has been demonstrated in healthy individuals whose self-reported valence and arousal measures correlated with physiological measures [22, 23] and is relevant to ED populations that have high levels of alexithymia [24]. Subjective ratings of images are frequently used to guide therapeutic practice and as salient stimuli for research studies, so understanding relationships among subjective emotional ratings and their objective physiological and neural response correlates is important for both therapeutic and research endeavours.

## Scope and inclusion criteria

This narrative review compares emotional responses to images of food and of weight/shape among subjects with ED in comparison with healthy controls, measured by subjective ratings, physiological arousal, and neural responses. The aim was to summarise the findings; provide clarification on outcomes; examine associations across emotional, physiological, and neuroimaging responses to ED-relevant stimuli; assess which stimuli result in the greatest responses in people with ED and explore the implications for treatment. Studies looking at participants diagnosed with or reporting symptomology of AN, BN, BED, and atypical variants previously diagnosed as “eating disorder not specified”, now termed “other specified feeding and eating disorders” were included [25]. Studies looking at participants with ED symptomology were included, but studies looking at participants with avoidant restrictive food-intake disorder were excluded. All ages, genders and ethnicities were included.

Emotional ratings for stimuli are gained by self-report through all studies used. Studies of peripheral autonomic physiological responses were included but research examining hormonal changes or changes in salivation was not. Physiological response is examined through changes in the startle-blink reflex, facial-electromyographic activity (EMG), skin conductance response (SCR), heart rate (HR) and posturographic destabilisation. Neural imaging data were gained by examining functional magnetic resonance imaging (*f*MRI) whilst participants viewed salient images. Only studies using *f*MRI were used to assess neural responses as this form of imaging is able to demonstrate reward activation, unlike event related potentials that only show cortical activation. Additionally, a lack of studies using alternative imaging forms was found.

Studies were found by using the databases Pubmed and Google Scholar. The search terms used for finding papers were: eating disorder, anorexia nervosa, bulimia nervosa, binge eating disorder, physiological, emotional, neurological, or neural, visual stimuli, food images or stimuli, body image or stimuli, shape image or stimuli, weight image or stimuli.

Twenty-nine papers were included in the review and these are considered in two sections: food (Table 1) and body/weight images (Table 2).

**Table 1:** Summary of physiological responses to food stimuli

	Participants	Image type	Physiological outcome	Result of food stimuli
Racine et al. 2016 [26]	• 19 AN females	• Positive images • Negative images • Neutral images • Food images	• Startle blink response	• Potentiated startle response > neutral
Friederich et al. 2006 [27]	• 15 AN female • 15 BN female • 30 HC female	• Food images • Body images • Positive images • Neutral images • Aversive images	• Startle blink response	• BN startle inhibition > neutral • No significantly different response in AN
Mauler et al. 2006 [28]	• 32 BN women • 32 HC women	• Unpleasant images • Neutral images • Pleasant images • Food images	• Startle blink response • Corrugator EMG • HR • SCR	• BN potentiated startle > control • BN increased corrugator activity > HC
Drobes et al. 2001 [29]	• 23 controls • 13 with restrained eating • 19 with binge behaviours • 21 food deprived subjects	• Unpleasant images • Pleasant images • Food images • Neutral images	• Startle blink response • HR • SCR	• Binge group potentiated startle response • Binge group greater startle response > control • Elevated HR for both binge and restrained
Suissa Rocheleau et al. 2019 [30]	• 18 controls • 19 with binge eating symptoms • 19 with dietary restriction symptoms • 23 with restriction and binge eating symptoms	• Food images • Pleasant images • Aversive images • Neutral images	• Startle blink response	• Higher self-reported craving correlated with a larger startle reflex • Binge and restrictive behaviours caused decreased startle reflex to food cues
Soussignan et al. 2009 [31]	• 16 AN females • 25 HC females	• Food images	• Facial EMG • SCR • HR	• AN decreased zygomatic activity
Racine et al. 2017 [32]	• 67 male and female participants who completed the eating disorder examination questionnaire	• Food images • Neutral images • Aversive images • Pleasant images	• Startle probe • Post auricular reflex • Facial EMG	• High eating disorder cognitions had increased corrugator activation > neutral • Increased post auricular reflex in those with binge eating behaviours > those without
Overduin et al. 1997 [33]	• 11 female restrained eaters • 13 female controls	• Neutral images • Food images • Body images	• SCR • HR • Startle reflex • Facial EMG	• Decreased corrugator activity in restrained > unrestrained eaters for food images • No other group differences seen
Leehr et al. 2016 [34]	• 16 Overweight women with BED • 23 Overweight women without BED • 22 Healthy controls	• High calorie food images • Non-food images	• Facial EMG	• Increased corrugator activator for all groups > non food
Svaldi et al. 2010 [35]	• 22 females with BED • 22 female controls with BMI over 25	• Food images	• HR and SCR	• Decreased HR and SCR > non-food in those with BED

**Table 2:** Summary of physiological responses to body stimuli

	Participants	Stimuli	Physiological outcome	Result of body shape stimuli
Oretga- Roldan et al. 2014 [36]	• 30 females with BN • 30 HC	• Image of own body • Startle noise	• Startle reflex • ECG • SCR	• Increased skin conductance response in BN > HC • Decreased startle reflex in those with BN > HC
Friederich et al. 2006 [27]	• 15 AN female • 15 BN female • 30 HC	• Food images • Body images • Positive images • Neutral images • Aversive images	• Startle blink response	• No startle reflex potentiation of body > neutral in either BN or AN
Herbert et al. 2013 [37]	• 35 healthy females screened for ED risk	• Body words • Neutral words	• Startle blink response • HR	• No difference in startle modulation between body and neutral words • HR differed significantly between body and neutral words • Correlation between women with high body dissatisfaction and increased startle response to body > neutral words
Spresser et al. 2012 [38]	• 53 females who took the eating disorder inventory	• Picture of participants own face altered to different levels of weight gain	• Startle blink response	• Startle responses to self-image predicted more variance in drive for thinness and body dissatisfaction scores
Vocks et al. 2007 [39]	• 28 female HC • 7 females with BN • 5 females with AN • 9 females with EDNOS	• Viewing own body image in a mirror	• HR • SCR	• Increase in SCR when viewing own body image in both HC and ED group
Overduin et al. 1997 [33]	• 11 female restrained eaters • 13 female controls	• Neutral images • Food images • Image of own Body	• SCR • HR • Startle reflex • Facial EMG	• No difference in EMG or startle reflex for body shape image • Increased SCR recovery time to body shape seen in both restrained and unrestrained eaters • Increased heart rate acceleration to body image in both restrained and unrestrained eaters
Forghieri et al. 2016 [40]	• 18 female HC • 10 females with AN • 3 females with BN • 4 females with EDNOS	• Neutral image • Looking at a mirror • Image of participants thin ideal body image	• Static posturography	• Increased body sway in ED group > HC when viewing own body image and ideal body image • Increased body sway for own body image and ideal > neutral image

### Food stimuli

#### Physiological responses

##### Startle-blink and postauricular reflexes

The startle-blink response increases when viewing threatening and unpleasant images and decreases while viewing pleasant images or exposed to appetitive cues [28, 41, 42]. The startle-blink reflex is also thought to infer a motivational state, with rodent studies showing a decreased startle blink to food and water [43]. In healthy controls, food images usually have appetitive properties and so should result in a reduced startle-blink reflex due to a motivational and pleasant response [28]; it was thought the opposite effect would be seen in those with ED.

In people with AN, food images produced similar startle-blink responses to those of positive and negative images, and were significantly different from neutral images, indicating that food images affected individuals with AN [26]. Food images also received arousal ratings similar to those of negative images [26], suggesting an association between physiological and emotional ratings to food images in those with AN, with a negative affect being seen. By contrast, a separate study, found that the startle-blink reflex to food images in people with AN was not significantly different from neutral cues or from those of healthy controls [27]. Food images were self-rated as highly anxiety provoking, demonstrating a disconnect between emotional and physiological responses in AN groups [27].

Mauler et al. looked at the startle-blink reflex in women with BN and healthy controls using positive, negative, neutral, and food images [28]. In those with BN, the startle-blink reflex for food images was significantly greater than for healthy controls. A potentiated startle-blink reflex for food images was also seen when compared to positive and neutral images within the BN group, suggesting that food images prompted negative affect in those with BN; this conflicted with verbal ratings of food images as pleasant and interesting in this group. However, when compared to the control group, those with BN rated food images as more disgusting and fearful, indicating that an association with physiological responses may be present [28]. Interestingly, when the startle-blink reflex was examined in a group with BN, no significant startle-blink response was seen [27]. Although, when subgroup analysis was performed for those with BN who were medication-free, a significantly reduced startle-blink reflex to food images versus neutral images was seen [27], indicating that food images may have evoked the appetitive motivational response; however, negative self-rated emotional responses to the same stimuli undermine this argument [27]. Additionally, the reduced startle-blink reflex suggests that selective serotonin reuptake inhibitors (SSRI) attenuate the startle blink response.

This difference in emotional ratings was also observed in those exhibiting binge or restrictive eating patterns: those with binge eating behaviours rated food images as more pleasant than those with restrictive behaviours [29]. Participants with binge-eating behaviours exhibited a larger startle-blink reflex for food images than both control and restrained eaters, indicating a greater negative affect and aversive emotional reaction [29]. Similar responses were seen in those with BN [28], which is expected, given the overlap in diagnostic features. The conflict between self-rated emotional and physiological responses may be explained by the use of a non-clinical sample [29]. Subjects in this study were healthy volunteers with no prior ED diagnosis who were chosen according to their scores on the eating disorders inventory (EDI) and split into restrained and binge eaters. Given that these individuals do not have formal ED diagnoses and only show patterns of restraint and binging behaviour, results cannot be applied to a clinical population [29]. Nonetheless, these findings suggest that responses in healthy populations with ED behaviours are similar to those with an ED diagnosis.

In another non-clinical sample of women reporting dietary restraint and binge-eating symptoms, food images resulted in a decreased startle-blink reflex, suggesting a more appetitive than aversive physiological response [30]. This reduced startle-blink reflex correlated with a greater self-reported craving, supporting the appetitive response theory [27]. In contrast to the startle-blink reactions, the postauricular reflex is a muscular reaction that occurs behind the ear and is elicited by a pleasant, startling stimulus [44]. Similarly, the postauricular reflex was found to be significantly larger to food images relative to neutral images in a non-clinical cohort with binge eating, suggesting that food was experienced as positive by those who binge eat [32]. No correlations, however, were seen between the startle-blink reflex and valence or arousal ratings for images [30], meaning a difference may exist between “liking” and “wanting” responses to food images. “Wanting” and “liking” are distinct neural processes: wanting is the process of incentive salience attributed towards a reward, whereas liking is an affective state arising from pleasure and no motivation for reward is required to induce it [45].

In summary, startle-blink reflex research presents a very mixed result with both potentiated and decreased startle-blink responses seen to food images, so provides little clarity on the responses generated to these images. However, the physiological response generated, frequently opposed the self-rated responses to these images indicating a decoupling between these responses.

##### Facial-electromyographic activity

Facial electromyographic activity (EMG) allows direct examination of facial responses to images by measuring muscle change and so is a physiological marker of emotional valence [33]. Increased corrugator activity is seen in response to frowning whereas zygomatic activity is seen with smiling [28, 33].

Individuals with AN had less zygomatic muscle activity when viewing food images than healthy controls, indicating reduced smile activity to these images [31]. Food pictures were rated as less pleasant by this group, so here a similarity between emotional and physiological response was seen [31].

In people with BN, viewing visual food cues resulted in significantly increased corrugator muscle use compared to pleasant images; this was not seen in controls [28]. A larger startle-blink reflex was also seen in this BN group, reinforcing the proposition that food images result in negative affect. These images were rated as pleasant; given that both physiological measures have the same aversive result, the lack of association between emotional and physiological responses is emphasised [28]. This increased corrugator activity to food images was also demonstrated in those with high eating-disorder cognitions compared to low on the eating disorder examination questionnaire, this was correlated with reduced pleasantness ratings of food within this group [32]. In restrained eaters both food and neutral images resulted in reduced corrugator activity, suggesting a pleasant response to these images, these images elicited emotional responses of arousal and craving in both restrained and unrestrained eaters [33]. However, as this study was only performed in those with restrained eating and not a diagnosed ED, the conclusions that can be made from this are limited.

People with BED, together with overweight and normal weight controls, rated food stimuli on wanting and liking scales adapted from Berridge [34]. Individuals with BED rated food higher on these scales than overweight individuals and heathy controls [34]. This is in contrast to the EMG data, where food stimuli evoked greater aversion for all groups, demonstrated by increased activation of the corrugator [34].

In summary, EMG data provide mixed physiological responses for food images, except for congruent negative responses in those with AN. For BN and BED, the picture is more complex, with food images inducing negative physiological responses in opposition to the self-rated pleasant scores.

##### Heart rate and skin conductance response

The skin conductance response (SCR) is an indirect measure of sympathetic nervous system activity, with the size of the response seen related to the level of arousal [23]. Heart rate (HR) is also a measure of autonomic arousal, with a deceleration in HR often seen for affective stimuli [23]. SCR and HR have been found to increase when smoking addicts were presented with smoking-related cues, but only SCR was found to increase in those with alcohol addiction [33]. However, there is mixed evidence as to whether a fear/phobic response results in HR deceleration or acceleration [46], making it difficult to interpret the response from this measure. Heart rate variability (HRV) is thought to be an important measure of emotional regulatory ability, and differences in HRV correlate with the capacity to regulate emotional responses [47]. HRV has also been found to be an index of self-regulatory strength, which is important for adaptive behaviour, and increased HRV is typically seen as beneficial [48]. This illustrates the problem of using HR as a physiological measure of emotion, as studies frequently do not consider HRV and the impact this has on emotional regulation.

In people with BED, SCR and HR were lower when viewing high calorie as opposed to low calorie images, suggesting that food images may have had a potentially soothing effect, despite being viewed as forbidden [35]. In restrained eaters HR response to food images was greater than seen in controls with restrained eaters also rating these images as more pleasant [29]. SCR and HR in restrained eaters, binge eaters, controls, and food-deprived participants were greater for pleasant and unpleasant images than for neutral [29]. Increases in HR when viewing food images may have been due to elevated arousal given the pleasant nature of the images, suggesting that an association may exist between physiological and emotional response. Alternatively, increased HR may represent fear or anxiety, suggesting a dissociation between self-report and physiological measures, and highlighting another limitation of HR as a physiological measure. Conversely, no difference in HR was seen between restrained and unrestrained eaters to food or non-food images, despite different emotional ratings for these images [33]. These negative findings could potentially be explained by the use of a non-clinical population, but this lack of change was also seen between those with BN and healthy controls across food, pleasant, unpleasant, and neutral cues [28]. This lack of association could be attributed to the short viewing window for images, with changes after 12 s no longer being measured [33]. As changes in HR due to emotion are typically slower, longer measurement durations are needed to capture them, particularly if the individual has low HRV.

In summary, in those with BED, high-calorie food images may be soothing due to the decreased HR and SCR seen. The lack of response frequently demonstrated in people who exhibit restrained or binge-eating behaviours makes general conclusions difficult.

#### Neural response studies

When viewing food images, activation was seen in the anterior cingulate cortex (ACC), dorsolateral prefrontal cortex (DLPFC) and medial orbitofrontal cortex (OFC) in people with AN, but not in healthy controls [49–51] (Table 3). Activation of the DLPFC and ACC is suggestive of an attempt to control and restrict an emotional and appetitive response due to the role these areas play in cognitive control [49, 52], and may accord with the restrictive eating patterns typically seen in this group. An emotional impact to these images can be inferred from the neural activation seen, as the medial OFC and ACC are involved in processing of emotional information [50]. This finding is supported by activation present in the DLPFC, which is implicated in emotional processing [49]. When people with anxiety were shown emotional stimuli, activation was seen in the DLPFC [52], so food stimuli may have generated a similar negative emotional reaction in those with AN. Despite a response that may suggest negative emotion, increased amygdala activation compared to controls was not seen for food images. Amygdala activation is seen in response to threatening stimuli [53], so it is unexpected that food images did not produce this response given the aversive emotional and physiological response previously reported. The fear network involves activation of the amygdala, PFC, ACC, insular cortex, striatum, and hippocampus, and so may be activated in those with AN to food stimuli, but this is unclear due to the lack of amygdala activation [54].

**Table 3:** Neural responses to food stimuli in people with AN

	AN Participants	HC participants	Stimuli	AN > HC	AN < HC
Uher et al. 2004 [50]	16	19	Colour pictures of food > non-food images Emotional aversive > neutral images	M.OFC, ACC	LI parietal, L occipital, posterior cerebellum
Brooks et al. 2011 [49]	18	24	Food > non-food images	DLPFC, cerebellum, R precuneus	Bilateral insula
Kim et al. 2012 [51]	18	20	High calorie food images > non-food images	L anterior insula, superior frontal gyrus, ACC, cerebellum	n/a

*R* right, *L* left, *DLPFC* dorsolateral prefrontal cortex, *OFC* orbitofrontal cortex, *ACC* anterior cingulate cortex, *M* medial, *S* superior, *I* inferior

These studies also demonstrate that activation of top-down control may be present to food images, with top-down control playing a role in response inhibition to stimuli [55]. This may help explain the restrictive behaviour shown towards food by those with AN.

In people with BN, activation of the ACC, medial OFC and DLPFC occurred when viewing food images [49, 50, 56] (Table 4), suggesting these stimuli had an emotional impact and, given DLPFC activation, this may be interpreted as a negative emotional response [49]. Activation of somatosensory and motor areas was also shown to be increased in this group compared to healthy controls due to activation of the supplementary motor area, precentral gyrus and caudate [49], pointing towards an appetitive response also being present. However, hypoactivation of these areas was also seen in those with BN and a high severity of binge eating [57]. So, though there is uncertainty about the somatosensory and motor response, there is evidence that differences may be accounted for by symptom severity. Activation in the ACC and insula are suggestive of an appetitive response, and this correlated with increased arousal ratings to food [56]. There is evidence that the insula is involved in perceiving the salience of signals, which leads to a subjective state of arousal [56, 58], so activation of the insula in response to food images should correlate with a physiological response indicating arousal. This has been seen in those with binge-eating characteristics, a feature of BN, suggesting that activation of these areas indicates arousal. Hypoactivation of the visual and occipital cortex was present in people with BN compared to controls, suggesting attention may be being diverted away from food stimuli [49, 50], correlating with a negative emotional response and indicating an aversive response. However, this contrasts with increased arousal findings, so increased insular activity may be due to its primary gustatory role, rather than the arousal of these images.

**Table 4:** Neural responses to food stimuli in people with BN

	BN participants	HC participants	Stimuli	BN > HC	BN < HC
Uher et al. 2004 [50]	10	19	Colour pictures of food > Non-food images Emotional aversive > neutral images	ACC, MOFC	L lateral and anterior parietal, LI parietal, L occipital, posterior cerebellum
Schienle et al. 2009 [56]	14	38	High calorie food images > disgust inducing images> neutral images	Insula and ACC	n/a
Brooks et al. 2011 [49]	8	24	Food > non-food	DLPFC, postcentral gyrus, caudate, precentral gyrus, supplementary motor area	bilateral superior temporal gyrus, bilateral insula, and visual cortex
Spangler et al. 2011 [59]	12	12	Computer generated thin and large body image Scrambled image	Pregenual ACC	n/a
Kim et al. 2012 [51]	20	20	High calorie food images > non-food images	Anterior Insula, R middle frontal gyrus and cerebellum	n/a
Skunde et al. 2016 [57]	28	29	Visual food stimuli > household item stimuli	No differences seen	No differences seen

*R* right, *L* left, *DLPFC* dorsolateral prefrontal cortex, *OFC* orbitofrontal cortex, *ACC* anterior cingulate cortex, *M* medial, *I* inferior

Insula and ACC activity also play a crucial role in the salience network, this network identifies relevant salient stimuli in order to guide behaviours [60]. This increased insula activity compared to HC for food images may suggest enhanced salience responses to food imagery in those with BN which coincides with the increased attention shown to these images.

Activation of the OFC as well as the ACC in those with BED, suggested an appetitive response to food stimuli occurs [56, 61] (Table 5). The OFC is the secondary gustatory cortex and so activation is expected in response to food images. It is also thought to be involved in reward sensitivity [56], with increased OFC activity potentially suggesting greater food motivation [62]. In people with BED, increased reward sensitivity to food images was self-reported so these findings correlate with neural responses. However, due to the limited studies available for BED it is difficult to draw firm conclusions.

**Table 5:** Neural responses to visual stimuli in people with BED

	BED participants	HC participants	Stimuli	BED > HC	BED < HC
Schienle et al. 2009 [56]	17	38	High calorie food images > disgust inducing images > neutral images	MOFC	n/a
Geliebter et al. 2015 [61]	10	10	Food > non-food images Food > non-food spoken word	Dorsal ACC	n/a

*ACC* anterior cingulate cortex, * MOFC* medial orbito frontal cortex

#### Summary of food stimuli

For food images, a dissociation exists between self-reported and physiological response in people with ED. This dissociation is present across a range of physiological measures and across ED pathologies. A difference in emotional response to images across ED diagnoses exists, with food images rated as more pleasant by those with BN and BED compared to those with AN. This is in contrast to the physiological response, which appears to be relatively consistent across all three groups that display aversive physiological responses to food images. The neuroimaging findings support the dissociation with negative emotional processing seen in those with AN, whereas motivational and appetitive responses appear to dominate in BN and BED.

### Body shape and weight

#### Physiological response

##### Startle-blink reflex

There is a dissociation across ED diagnoses between emotional responses and the startle-blink reflex. When people with BN viewed a video of their own body, they had a smaller startle-blink reflex than that of healthy controls, despite those with BN having rated these images as more unpleasant [36]. A reduced startle-blink reflex for those with BN was also present during a noise-only trial with no image stimulus, which may be interpreted as a sense of alertness throughout the trial due to the knowledge that they would be viewing images of their own body at a later point [36]. This is reinforced by the elevated attention paid to these trials in attentional bias studies by this patient group [5, 63]. However, the startle-blink reflex when viewing own-body image for those with BN was smaller than the noise-only trial. This finding cannot be explained by vigilance and is contrary to the emotional ratings where own-body image evoked unpleasant feelings and greater arousal [36], suggesting a dissociation between emotional and physiological responses. A further study in those with BN observed no change in the startle-blink reflex between viewing body-shape and neutral images, despite high anxiety ratings towards the images [27]. This pattern did not change once those taking SSRI were excluded, unlike with food images [27]. These findings are replicated in people with AN, with no change in the startle-blink reflex observed for body-shape images, despite similarly high anxiety ratings [27].

In healthy women screened for their risk of ED, no difference was seen in the startle-blink response between body and neutral words [37]. However, an association was found with women who reported high body dissatisfaction, but not drive for thinness or BN, and a larger startle-blink response for body shape compared to neutral words. Body-shape words were rated as more negatively valenced and higher for arousal, with significantly greater differences in ratings seen for those with higher EDI scores [37]. Emotional ratings and startle-blink responses were recorded in response to self-photos that had been edited to represent a large and XL shape [38]. XL-shape images resulted in a startle-blink response that significantly predicted more variance in EDI body dissatisfaction and drive for thinness scales than emotional self-report. No significance was seen for the BN subscale rating [38]. In line with previous studies of the differences between self-report and physiological data, these results suggested that the startle reflex response may be a better predictor of ED behaviour than self-reported emotional ratings, which have potential reporting bias [38].

In summary, startle-blink reflex studies show little change between neutral and body-shape images, despite these images being consistently rated as more unpleasant. However, the startle-blink reflex was found to be a better predictor of ED pathology than self-report in one study, so further research is needed to explore this discrepancy.

##### HR, SCR and posturographic destabilisation

In women with BN, a larger SCR was seen compared with healthy controls when viewing a video of their own body [36]. Unlike startle-blink responses [36], this correlated with more unpleasant feelings and arousal in this group, which suggests an aversive response was present in those with BN to images of self. Despite those with BN demonstrating greater negative emotions across a greater range of cognitions compared with healthy controls, no significant difference in SCR was seen in those with ED compared with healthy controls when looking at their own body in a mirror [39]. An association was seen between the physiological and emotional reaction, as SCR increased when looking at self in the mirror in this group [39]. However, this increase in SCR was the same as in healthy controls, so the value of this association is decreased.

In both restrained and unrestrained eaters, self-image resulted in greater negative arousal, as longer SCR recovery times were seen [33]. However, there was no difference in SCR between these groups and no difference in emotional rating was seen between restrained and unrestrained eaters [33]. This points towards the response to body-shape images being aversive to those with normal eating patterns as well as those with disordered eating, and would explain the lack of significant effect seen in the startle-blink reflex [33].

A potentiation of the cardiac defence response is seen with a HR acceleration-deceleration pattern found in those with BN when exposed to own-body images [36]. The same response was also seen in both restrained and unrestrained eaters when shown pictures of self [33]. This pattern suggests an aversive response occurs coinciding with the negative emotional ratings reported towards these images [36].

Postural control is affected by psychological states, with anxiety mediated by visual stimuli resulting in increased body sway [40]. A group of patients with ED and healthy controls were shown their own image reflected in a mirror as well as the image of a thin ideal model [40]. The ED group experienced greater body sway when looking at both images compared with the healthy controls, representing postural destabilisation [40]. This suggests that, in those with ED, images of self and a thin ideal are anxiety provoking and may induce a quasi-phobic state that is not seen in healthy controls.

In summary an aversive physiological response is present in those with ED to body-shape stimuli based on their autonomic responses; This correlates with negative self-rating of the same images.

#### Neuroimaging response

In those with AN, neuroimaging evidence supports an association between emotional and neural response. In females with AN, increased amygdala activation was seen to body-shape and weight stimuli [53, 64–66] (Table 6). These neural responses correlated with self-reported negative affect towards these images [37] and correspond with responses in previous emotional and physiological studies [36]. Amygdala activation is also correlated with unpleasantness ratings of XL-shape images of self, reinforcing the association between emotional and neural response in people with AN [53]. Along with amygdala activation, activation of other brain areas involved in the fear network was also seen [54] suggesting that this network is activated when body images are viewed. Activation in areas known to have a role in emotion: medial-prefrontal cortex (PFC), DLPFC and limbic system, was demonstrated to self-images, supporting the finding of a negative emotional response [52, 53, 64, 67, 69]. Right cingulate hyperactivation, an area directly involved in negative emotional processing, was also found when those with AN reported negative affect towards these images reinforcing the association between neural and emotional responses [65].

**Table 6:** Neural responses to body shape stimuli in people with AN

Author	AN Participants	HC participants	Stimuli	AN > HC	AN < HC
Seeger et al. 2002 [66]	3	3	Distorted image of self > distorted image of other > abstract colour image	R amygdala, brainstem, R gyrus fusiformis	n/a
Wagner et al. 2003 [67]	15	11	Distorted image of self > distorted image of other > abstract colour image	DLPFC and intraparietal sulcus	n/a
Uher et al. 2005 [68]	13	18	Line drawing of body shape > line drawing of houses	n/a	R parietal, R and lateral fusiform gyrus
Sachdev et al. 2008 [69]	10	10	Images of self > grey screen	n/a	Insula, PFC and occipital lobe
			Image of other > grey screen	MPFC	n/a
Miyake et al. 2010 [64]	24 total 12 restrictive 12 binge purge subtype	12	Negative body image words > neutral words Negative emotional words > neutral words	Amygdala, L MPFC, DLPFC and LI parietal	n/a
Vocks et al. 2010 [65]	13	27	Self-image	n/a	LI parietal lobe, M temporal gyrus, L uncus, parahippocampal
			Image of other	R amygdala, bilateral para hippocampal, L uncus, L hippocampus, R posterior cingulate	n/a
Miyake et al. 2010 [53]	22	11	Distorted own body image	Amygdala, MPFC, DLPFC	n/a
			Distorted image of other	R amygdala	
Castellini et al. 2013 [52]	18	19	Own body image normal > distorted	frontal gyrus, M temporal gyrus, DLPFC	n/a

*R* right, *L* left, *DLPFC* dorsolateral prefrontal cortex, *OFC* orbitofrontal cortex, *ACC* anterior cingulate cortex, *M* medial, *S* superior, *I* inferior

Areas of hypoactivation compared to controls were demonstrated. These were mainly in the lateral PFC and inferior parietal lobe [50, 65, 68, 69], which were interpreted by the author as decreased capacity for attention being present, and that decreased visual processing may be occurring in an attempt to avoid stimuli with a negative emotional impact [65]. Hypoactivation of these areas also suggests decreased bottom-up processing of these images as well as increased top-down control with activation of the PFC, which may be due to the aversive nature of these stimuli [55].

In contrast to *f*MRI studies of AN groups, only one study demonstrated increased amygdala activation in response to body-shape stimuli in individuals with BN [64] (Table 7), suggesting that these stimuli were less threatening to those with BN. However, as activation of the ACC and ventromedial PFC—centres involved in the processing of emotional information—was seen, an emotional response was still evoked to body-shape images [59, 64]. The negative emotional properties of these neural activations were confirmed by activation of the limbic system in conjunction with self-reported negative emotional affect to these images [65]. Once again, increased top-down processing is seen to body-shape images suggesting an aversive nature and inhibition of focus towards these images [55].

**Table 7:** Neural responses to body shape stimuli in people with BN

	BN participants	HC participants	Stimuli	BN > HC	BN < HC
Uher et al. 2005 [68]	9	18	Line drawings of body shapes > line drawing of houses	n/a	R and lateral fusiform gyrus
Miyake et al. 2010 [64]	12	12	Negative body image words > neutral words Negative emotional words > neutral words	R amygdala, L ventro MPFC	n/a
Vocks et al. 2010 [65]	15	27	Own body	n/a	RI parietal, right middle frontal
			Image of other	Bilateral postcentral gyrus and bilateral hippocampal	n/a
Miyake et al. 2010 [53]	11	11	Own distorted body image	R occipital and R parietal	MPFC, DLPFC, amygdala
			Distorted body image of other	R occipital and R parietal	n/a
Spangler et al. 2011 [59]	12	12	Distorted image of self > distorted image of other > abstract image	Pregenual ACC	n/a

*R* right, *L* left, *MPFC* medial prefrontal cortex

#### Summary of body-shape stimuli

Viewing self-image resulted in an increase of negative emotions across all ED diagnoses with no distinct variations seen, emphasising the importance of body shape and weight in ED maintenance and pathology. The physiological impact was mixed, with little clarity provided by the current evidence base. A lack of physiological response was seen in startle-blink reflex, whereas SCR and HR suggest aversive responses that are associated with negative emotional affect. In neuroimaging, a consistent aversive and negative emotional impact was seen. This suggests a consistent association across emotional, physiological, and neural responses to body image and is supported by findings of postural destabilisation.

## Methodological issues

Methodological issues may account for inconsistent physiological responses to both food and body-shape images. In some studies, participants selected the imagery used, whereas other studies provided images. Some studies obtained participant ratings of image salience, some studies used images selected for their valence by volunteers with both ED diagnoses and healthy controls [27, 65], and other studies did not measure the valence or salience of images. A lack of replication of findings was also present in studies using the same physiological measure, limiting the strength of the conclusions that can be formed. This lack of replication was particularly prevalent in neuroimaging studies with a wide range of activation areas indicated. A large overlap of activation areas with healthy controls was seen for body-shape stimuli and suggests that these results may be continuous between healthy individuals and those with ED.

SSRI attenuate the startle-blink reflex [43], and given that this was not controlled for in several studies [26, 29], the startle-blink reflex to food images may be underestimated and the validity of results weakened. SSRI have also been shown to have an impact on HRV and HR, and so may have attenuated other physiological measures [70, 71]. A decrease in HRV was seen in those with anxiety and depression taking an antidepressant compared with those who were not [71]. The same effect may be present in those with ED taking SSRI, which could impact capacity to regulate emotional responses. As use of SSRI was not consistently controlled for, this may invalidate the physiological responses seen: serotonin pathways have a role in both autonomic regulation and modulation of emotion [72], so use of SSRI has the potential to modulate both these behaviours, further propagating the dissociation between subjective and emotional responses.

Inconsistent levels of ED pathology across this literature also reduce the validity of results and limit comparisons. Several studies rely on participants presenting ED symptoms but no formal diagnosis, whereas clinical thresholds in other studies vary widely. The use of broad diagnostic categories can also lead to mixed results, since binge-eating behaviour can occur in some patients with AN.

The majority of studies only had female participants, despite binge eating prevalence being comparable in males and females [73]. This greatly reduces the applicability of these findings to the wider ED population, as males typically present with less shape and weight concern, so the responses to these stimuli may be very different from females and need to be examined separately [74].

## Conclusions

This narrative review draws together emotional, physiological, and neural responses that studies have demonstrated in using food and body-shape/weight stimuli in people with ED. The pattern for food stimuli is inconsistent, with differing responses seen to these stimuli amongst the ED pathologies and a dissociation across the emotional, physiological, and neural modes of response measurement. Body-shape and weight stimuli induce more consistent emotional, physiological, and neural responses across all the ED groups than food stimuli, with greater associations seen across all three modalities.

People with AN have mixed responses to food images. Food images are consistently rated as anxiety provoking and generate a negative emotional impact. This was seen in the neuroimaging findings, with activation of areas indicating negative emotional processing; however, no amygdala activation was seen, which would be expected given the high anxiety ratings. The physiological response is also quite varied with some studies indicating an aversive response is present, while in others no effect was seen for the stimuli.

Body-shape and weight stimuli consistently induce a negative emotional self-reported affect, produce an aversive physiological response, and result in activation of areas associated with processing negative emotions. Additionally, amygdala activation was observed to these stimuli in people with AN, indicating that these stimuli do not just evoke negative emotions but are also perceived as threatening to the individual. However, this association is weakened by the physiological response findings: several startle-blink studies found no significant differences in the startle-blink response, so this association relies on data from HR, SCR, and posturographic destabilisation research.

In individuals with BN, a dissociation exists in the emotional, physiological, and neural response. Aversive physiological responses were seen in both startle-blink and facial EMG to food stimuli, despite often being rated as pleasant. This was also seen in the neural response, with activation in areas associated with processing negative emotion. Further neural activation was seen, suggesting an appetitive response associated with greater arousal ratings, but this was not present in physiological studies.

Conversely for BED, an association does appear to exist but only between the neural and emotional response. A motivational and reward response was seen in neuroimaging that correlated with food images being consistently rated as more pleasant and with higher reward. However, this appetitive response was not seen physiologically, where an aversive response appears to be present as demonstrated by increased startle-blink reflex and corrugator activity. Unfortunately, this cannot be compared across all three measures for those with BED due to a lack of appropriate neuroimaging studies comparing individuals with BED to healthy controls.

### Associations across neural, physiological, and subjective emotional responses

In healthy individuals, there are several proposed mechanisms for the associations across neural, physiological measures, and emotional responding. HRV, which was earlier discussed as a measure of emotional regulation, has been found to be correlated with the medial-PFC activation during emotional activation [75]. Further correlation for HRV was also found to the caudate nucleus, the mid-left insula, and the periaqueductal grey matter [75]. These areas are part of the medial visceral network, indicating that this pathway is involved in the emotional and cognitive control of the autonomic response. The ventro-medial PFC has also been further implicated in controlling cardiovascular responses to emotional stimuli [76], with problems in the ventro-medial PFC shown to cause altered responses to these stimuli, which may be the case in those with ED. Substantial evidence also exists to support the role the amygdala plays in controlling autonomic nervous system responses to fear stimuli with increased amygdala responses correlating with both increased SCR and HR [77, 78].

Abnormal amygdala responses to fearful stimuli have been shown to be present in several psychiatric disorders [79], this may also be the case in ED, and offers an explanation for the dissociated responses seen due to the role the amygdala plays in autonomic control. Another possible explanation is the effect that physiological impacts of ED have on neural function, with malnutrition resulting in widespread changes throughout the brain and in some instances causing decreased grey matter [80]. Altering of the OFC and insula also appears to take place in ED, suggesting that altered reward processing is present [80]. Given the role neural function plays in autonomic control of emotions it is logical that this could lead to a decoupling of the subjective and objective responses in people with ED that impact neural structures.

There are several other possible explanations for the lack of association across neural, physiological, and subjective emotional responses frequently seen in those with ED. The high rates of alexithymia that are present in those with ED might result in a decoupling between self-reported emotional responses and physiological responses. This decoupling was demonstrated when those with alexithymia were put under the stressful scenario of giving an impromptu speech [81]. Given that food and body-shape images may induce negative feelings and stressful responses, a similar decoupling response may have been present in these studies. Another possible explanation is that emotional responses are not always coherent, even within a healthy population [82], so the differences seen may be due to normal emotional responses within the population. The motivational conflict hypothesis offers an additional explanation for the lack of association seen for those with binge-eating behaviours by suggesting that the eating behaviours seen are associated with both positive and negative reactions towards food [32]. So it may be this mixed response results in both positive and negative subjective and objective responses. Blunting of the startle-blink reflex in those with AN to fear stimuli offers might explain why no potentiation of the startle-blink reflex was frequently seen [83]. A lack of association between subjective and objective responses to affective images was demonstrated in those with AN, so the lack of correspondence may not be cue dependent but instead point to a more general dissociation seen in those with AN [84]. There is potential that this may also be the case in other ED offering an explanation for the differing results reported in this review.

### Implications for clinical practice

The associations and dissociations described in this review have implications for clinical practice. Images are increasingly being used in both VR [7] and imagery-based CBT [6]. In order for these treatment methods to be effective, the impact these stimuli have on participants needs to be understood. The subjective ratings of the patient and their objective responses often oppose each other, with stimuli inducing an aversive or motivational self-report response but having the opposite impact, or no impact, physiologically and neurally. Substantial negative impact was found for self-image across all modalities, which is particularly important for mentalisation practices. Food stimuli may be less appropriate to use in therapy and may require careful consideration for research due to the differing responses seen and inconsistency in impact. In VR, repeated food image exposure has been used in order to decrease negative emotions [85], but in those with BN and BED, motivational and appetitive responses were shown to these images alongside negative affect, suggesting some dissonance in responses.

Research has shown that alexithymia has a negative impact on treatment outcomes, with poorer responses to therapeutic interventions seen [86]. This may be due to the decoupling that is seen in alexithymia, with a decreased awareness of emotional cues and responses potentially reducing the ability to engage successfully in psychotherapy [87]. This decoupling is similar to the lack of association present between the subjective and objective responses. Those patients who display a high lack of association, therefore, may also have worse treatment outcomes and be less successful in therapeutic engagement. Thus, the findings of this review have important implications for treatment outcomes.

In order to increase the validity of studies, images are initially shown to a group of patients who rate the images to ensure that they are salient. However, given that there may be a dissociation between these subjective emotional ratings and the objective physiological and neural responses, the images selected may not have the desired impact. This needs to be considered when using imagery in trials, as it may impact findings. Motivational conflict present may also have important treatment indications as it means both positive and negative feelings or reactions towards food/body-shape need to be treated and addressed by clinicians [32].

In summary, body-shape and weight stimuli appear to consistently generate more aversive responses in people with ED than food stimuli, suggesting that these images may have greater salience for patients with ED. Given the increased consistency between subjective and objective responses to these images, greater reliance can be made on self-reported responses to these stimuli when considering their use in therapy and research.

### Limitations

Several limitations are present in the existing literature. The majority of studies only focused on females and as evidence exists that men and women differ in their response to emotional stimuli [88], research reported in this review may not be generalisable. In addition, there was a large heterogeneity in the stimuli used, the physiological measures and the emotional valence ratings that reduces the validity of this review’s findings. This, combined with the lack of comparison across all three measures in the majority of studies, provides a significant limitation to the strength of conclusions that can be drawn. The small sample sizes consistently used in studies using *f*MRI also greatly limits the validity of their findings.

Food and body-shape importance depends on individual cultural beliefs, and this was not always considered in studies. Body-shape studies were mainly focused on self-image, so the impact of images of others is still relatively unknown. Due to the lack of response seen in startle-blink studies for body-shape stimuli, the physiological findings are limited to HR and SCR, which are less reliable measures. This review is further limited by the measures that were focused on: by only examining neural imaging studies using *f*MRI, other neuroimaging measures were excluded. Several physiological measures were also excluded, such as hormonal changes, and the responses seen in these measures may differ from the conclusions drawn in this review.

## Data Availability

Not applicable.

## Abbreviations

ACC: Anterior cingulate cortex
AN: Anorexia nervosa
BED: Binge eating disorder
BN: Bulimia nervosa
CBT: Cognitive Behavioural Therapy
DLPFC: Dorsolateral prefrontal cortex
ED: Eating disorders
EDI: Eating Disorders Inventory
EMG: Electromyographic
fMRI: Functional magnetic resonance imaging
HR: Heart rate
HRV: Heart rate variability
OFC: Orbitofrontal cortex
PFC: Prefrontal cortex
SCR: Skin conductance response
SSRI: Selective serotonin reuptake inhibitor
VR: Virtual reality
